# Frontotemporal Dementia-Associated N279K Tau Mutation Localizes at the Nuclear Compartment

**DOI:** 10.3389/fncel.2018.00202

**Published:** 2018-07-12

**Authors:** Maxi L. Ritter, Jesús Avila, Vega García-Escudero, Félix Hernández, Mar Pérez

**Affiliations:** ^1^Departamento de Anatomía Histología y Neurociencia, Facultad de Medicina, Universidad Autonoma de Madrid (UAM), Madrid, Spain; ^2^Centro de Biología Molecular Severo Ochoa, Consejo Superior de Investigaciones Científicas (CSIC), Universidad Autonoma de Madrid (UAM), Madrid, Spain; ^3^Centro de Investigación Biomédica en Red sobre Enfermedades Neurodegenerativas (CIBERNED), Carlos III Institute of Health, Madrid, Spain

**Keywords:** FTDP-17, Alzheimer, tau, transport, nucleus

## Abstract

Tau is a microtubule-associated protein that plays an important role in Alzheimer’s disease and related tauopathies. Approximately one-half of all cases of Frontotemporal dementia with parkinsonism-17 (FTDP-17) are caused by mutations in the MAPT gene. The N279K mutation is one of the three mutations more prevalent in FTDP-17 cases. Several studies have demonstrated that N279K Tau mutation alters alternative splicing inducing the presence of exon 10. Tau is mainly found in the cytosol of neuronal cells although it has also been localized within the nucleus. Here we demonstrate by biochemical and immunohistochemistry studies in COS-7 cells, that the proportion of mutant N279K Tau increases compared with wild-type at the cell nucleus although cell viability is not affected. These data will provide us with a better outline of the nuclear role of tau protein offering new clues related with this tauopathie.

## Introduction

Tau protein is composed of six different isotypes generated by alternative splicing mechanisms in the central nervous system (CNS) of mammals. Three of these isoforms contain three copies of the microtubule-binding domain (Tau3R) whereas the other three isotypes contain four repeats (Tau4R). The expression of some of these Tau isoforms is developmentally regulated. Thus, mouse isoforms lacking exon 10 (Tau3R) are found at early developmental stages whereas Tau isoforms containing exon 10 (Tau4R) are mainly found in murine neurons at mature developmental stages (Avila et al., 2004; Sergeant et al., 2005). In adult human brain both Tau 3R and Tau 4R are present, although in newborn neurons such as those in the hippocampal dentate gyrus Tau 3R is the main isoform (Bullmann et al., 2007).

Some Tau gene mutations alter proportion Tau4R/Tau3R and this alteration is pathological. The mechanisms regulating the ratio of Tau4R/Tau3R are due to mutations altering splicing in some frontotemporal dementia patients present in exon 10 or intron regions flaking that exon, mainly close to the 5′ splice site of exon 10. N279K mutation (SNP ID number: rs63750756) present in exon 10 is extremely rare among healthy individuals, but it is among most frequent causes of familial frontotemporal dementia^1^. At molecular level, N279K mutation affects exon 10 splicing allowing exon 10 to be incorporated more frequently and causing an increase of Tau4R isoforms (Delisle et al., 1999; Dawson et al., 2007). The N279K mutation strengthens a poly-purine positive cis-element present within exon 10, resulting in increased exon 10 inclusion during splicing process (Hutton, 2001). Consequences of that alteration in Tau 4R/3R proportion is the disruption of subcellular vesicle trafficking and induction of cellular stress at least in iPSC-derived neural stem cells (Wren et al., 2015).

Tau has mainly an axonal localization and can be found associated with microtubules although that interaction is highly dynamic explaining why tau can also be present in other cellular compartments (Janning et al., 2014). Thus, Tau can be also associated with the plasma membrane (Brandt et al., 1995) in an interaction that could be modulated by Tau phosphorylation (Arrasate et al., 2000; Gauthier-Kemper et al., 2018). Also, the presence of a nuclear antigen reacting with several Tau antibodies has been reported, mainly in proliferating cells, demonstrating that Tau is also a nuclear protein (for a review see Bukar Maina et al., 2016).

Nuclear location studies seems to confer Tau an important role in nucleolar structure conformation and heterochromatinization of ribosomal genes (Sjöberg et al., 2006; Rossi et al., 2008). The role that it might play in the nucleus and the physiological consequences derived from its interaction with DNA remains to be elucidated. However, a function related to protection of genomic integrity has been recently suggested to Tau protein (Sjöberg et al., 2006). Interestingly, Tau binding to DNA is modulated by phosphorylation (Camero et al., 2014). However, it is unknown how Tau protein is transported to nucleus as not clear import or export sequences has been described in Tau protein.

Here, we explored the nuclear localization of N279K isoform in order to know the effect of that mutation in nuclear localization. Our data demonstrate that the proportion of mutated Tau, at the cell nucleus, increases compared with wild-type. The consequences of that distribution are discussed.

## Materials and Methods

### Antibodies

List of antibodies used are shown in Table 1. The antibodies against Tau used in this study were: Tau 12 (N-terminal, mouse monoclonal); Tau 46 (C-terminal, mouse monoclonal); Tau 1 (unphosphatase-sensitive epitope corresponding to Ser199/202, mouse monoclonal); 7.51 (against microtubule-binding domain); AD2 (Tau phosphorylated at S396/S404, mouse monoclonal); AT-8 (Tau phosphorylated at S202, mouse monoclonal).

**Table 1:** List of antibodies.

Antibody	Epitope	Source, host species, catalog/clone/lot No.,	Dilution
Tau 12	Aminoacids 6–18 of human tau	Abcam; Mouse monoclonal; Cat# ab74137	1/500 (WB)
Tau 46	Aminoacids 404–441 of human tau	Abcam; human; Mouse monoclonal; Cat. #: ab22261	1/1000 (WB)
Tau 1	Unphosphorylated tau in amino acids 198–206	Calbiochem San Diego, CA, USA; Mouse monoclonal	1/5000 (WB); 1/500 (IF)
Tau 5	Amino acids 210–241 of bovine Tau	Abcam; Mouse monoclonal; Cat# ab80579	1/1000
7.51	The microtubule-binding region	Kindly provided by Dr. C. M. Wischik, Aberdeen, UK; Mouse monoclonal	1/100 (WB)
AD2	Phospho tau in S396/404	Biorad Laboratories; Mouse monoclonal; Cat# 56484	1/1000 (WB)
AT8	Phospho tau in S202	Innogenetics. Cat #90206	1/1000 (WB)
Anti-ERK1	C-terminus de ERK 1	Rabbit polyclonal: Santa Cruz Biotechnology; Cat# (C-16): sc-93	1/100 (IF)
Anti-β Actin	Slightly modified β-cytoplasmic actin N-terminal peptide, Ac-Asp-Asp-Asp-Ile-Ala-Ala-Leu-Val-Ile-Asp-Asn-Gly-Ser-Gly-Lys, conjugated to KLH.	Sigma-Aldrich; Monoclonal; Cat# A5441	1/5000 (WB)
Anti-GADPH	Rabbit muscle GAPDH	Abcam; Monoclonal; Cat# AB8245	1/3000 (WB)
Anti-laminin B1	A monoclonal antibody against the C-terminal of Laminin B1	Santa Cruz Biotechnology; Mouse monoclonal; Cat# sc-377000	1/100 (WB)

The monoclonal antibody directed against β-actin (Sigma St. Louis, MO, USA) was used as internal control for protein quantification. Anti-GADPH (Abcam) and anti-Lamin B1 (Santa Cruz Biotechnology) were used as internal control for nuclear extracts.

### Materials

The anti-proteases cocktail and Leptomycin B and the rest of the reagents were purchased from Sigma-Aldrich.

### Tau Expression Constructs

The largest CNS isoform of wild-type human Tau was expressed from the SV40 early promoter using the plasmid pSGT42 previously described (Montejo de Garcini et al., 1994). To engineer the N279K mutation, mutagenesis was carried out using the polymerase chain reaction (PCR) with primer N279K forward5′-GTGCAGATAATTAAGAAGAAGCTGG-3′ and primer N279K reverse 5′-CCAGCTTCTTCTTAATTATCTGCAC-3′ which include the mutated codon. The fragment generated by PCR amplification on pSGT42 using primers described above was digested with *BglII* and *EcoRI* and ligated into the eukaryotic expression vector pSG5 (Stratagene) under the control of SV40 early promoter digested with the same enzymes to obtain pSGTN279K.

**Figure 1:**
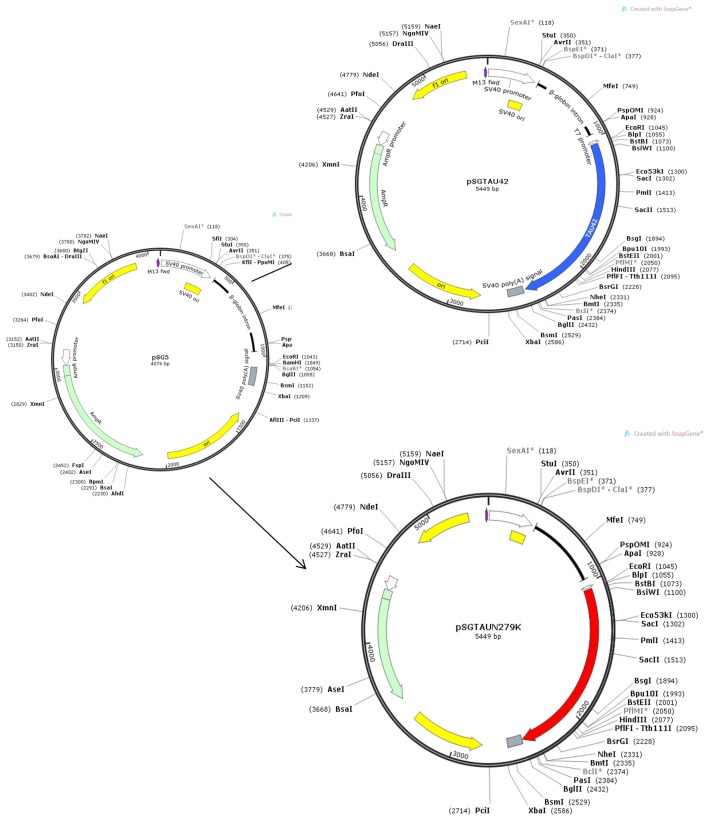
Maps of Tau constructs used in this work.

Positive clones were analyzed by restriction analysis to test for the proper orientation and correct size of the inserts. Finally, the constructions were confirmed by DNA- sequencing analysis. Figure 1 shows the maps of Tau constructs used in this work.

### Cell Culture and DNA Transfection

African green monkey kidney fibroblasts (COS-7) cells (Gluzman, 1981) were grown in Dulbecco’s modified Eagle’s medium supplemented with 10% (vol/vol) fetal bovine serum (FBS). Cells were transfected with the cDNA constructs using PEI reagent (Polysciences, Inc) according to the manufacturer’s instructions. The empty vector pSG5 was used to transfect control cells.

### Leptomycin B Treatment

One hour before the end of transfection, COS-7 cells were incubated in FBS-free DMEM containing vehicle (methanol) or 20 ng/ml Leptomycin B1.

### Toxicity Assays

Cell death was assayed by using the LIVE/DEAD viability/cytotoxicity kit (Invitrogen, Carlsbad, CA, USA) to label live cells and ethidium homodimer-1 to label dead cells. 1 × 10^5^ COS-7 cells were seeded to each well of a 24-well plate and transfected with the plasmids described above. After 48 h postransfection, cell viability was measured using LIVE/DEAD viability kit. Cells were incubated for 20 min with 2 μM propidium iodide and 1 μM calcein. After staining, live cells (green) and dead cells (red) were visualized on a Leica fluorescence microscope and images were taken. Three fields (selected at random) were analyzed per well (100–500 cells/field) and counted with ImageJ software. Cell viability was defined in each condition as the percentage of live cells vs. the total number of cells.

### Western Blotting

At 48 h post-transfection, cells were homogenized in lysis buffer (20 mM HEPES pH 7.4, 5 mM EDTA, 100 mM NaCl, 1% Triton X-100, 0.1 mM sodium orthovanadate, protease inhibitor cocktail and 0.1 μM Okadaic acid). Lysates were centrifugated at 10,000 *g* for 15 min at 4°C and protein samples were quantified by the BCA protein assay. Samples were separated on 10% SDS-PAGE and electrophoretically transferred to a nitrocellulose membrane (Schleicher & Schuell GmbH). The membrane was blocked by incubation with 5% semi-fat dried milk in PBS and 0.1% Tween 20 (PBSM), followed by 1-h incubation at room temperature with the primary antibody in PBSM. The following primary antibody dilutions were used: T12 (1/500); T46 (1/1000); Tau5 (1/1000): Tau 1 (1/5000); 7.51 (1/100); AD2 (1/500); anti-GADPH (1/3000); anti-Lamin B1 (1/250) and anti-β actin (1/5000). After three washes, the membrane was incubated with a horseradish peroxidase-anti-mouse Ig conjugate (DAKO), followed by several washes in PBS-Tween 20. The membrane was then incubated for 1 min in Western Lightning reagents (PerkinElmer Life Sciences).

Blots were quantified using the EPSON Perfection 1660 scanner and the ImageJ1.46r image analysis system. The levels of various markers were normalized to the β-actin present in each band.

### Nuclear Extracts

Adherent cells were washed with ice-cold PBS and scraped into ice-cold hypotonic Buffer A (20 mM HEPES pH 7, 0.15 mM EDTA, 0.015 mM EGTA, 10 mM KCl, 1% NP-40 supplemented with protease inhibitors), incubated for 30 min on ice in a rotating wheel and pelleted by centrifugation at 2300 rpm for 5 min at 4°C. Supernatant was collected as cytosolic fraction. Nuclear pellet was washed in five volumes of buffer B (10 mM HEPES pH 8, 25% (v/v) Glycerol, 0.1 M NaCl and 0.15 mM EDTA). After centrifugation as above, nuclei in the pellet were resuspended in two cellular volumes of Buffer A.

### Immunofluorescence and Confocal Microscopy

For immunofluorescence studies, cells were fixed with 4% formaldehyde. Subsequently, the fixed cells were permeabilized with 0.1% Triton X-100 and Glycine 1 M for 30 min. After fixation, the coverslips were blocked with 1% bovine serum albumin for 30 min and subsequently incubated with primary antibodies in PBS containing 1% bovine serum albumin for 1 h. Coverslips were rinsed three times with PBS and incubated 45 min with Alexa 488-conjugated anti-mouse (diluted 1:400; Thermo Fisher). All the coverslips were finally counterstained for 3 min with 4′,6-Diamidine-2′-phenylindole dihydrochloride (DAPI; 1:1000, Calbiochem-EMD Darmstadt, Germany). After washing with PBS, the coverslips were mounted with FluorosaveTM (Calbiochem, San Diego, CA, USA). Confocal images were obtained using a TCS SP5 Spectral Leica Confocal microscope using an oil-immersion 40× objective with sequential-acquisition setting. The detector pinholes were set to give a 0.3 μm optical slice.

### Data Analysis

Data are presented as the mean ± SD. Two group comparisons were made using unpaired Student’s two-tailed *t*-test. Significance was accepted at *p* < 0.05.

## Results

### Overexpression of Tau Without Cell Death

**Figure 2:**
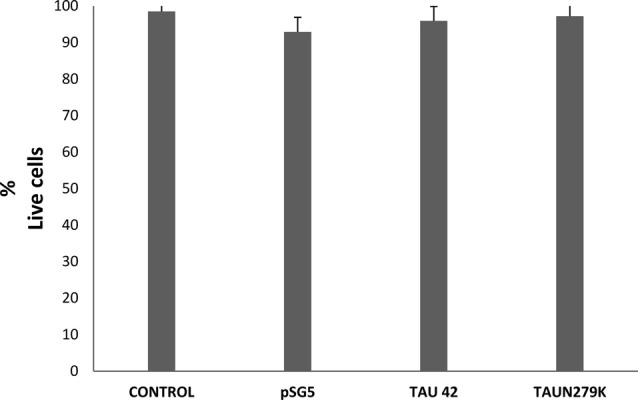
Cell viability of COS-7 cells. Cell viability under control conditions, transfected with empty pSG5 or with pSG5-Tau 42 or with pSG-Tau N279K was measured by labeling with calcein-AM (live cells) and ethidium homodimer-1 (dead cells), 48 h after transfection. Quantification of the percentage of live cells in each condition is shown.

To determine if overexpression of plasmids used in this work was or not toxic for the cells, we performed a toxicity assay. Figure 2 shows that cell viability under control conditions, transfected with empty pSG5 or with pSG5-Tau 42 or with pSG-Tau N279K plasmid did not correlated with cell death, given the low rate of cell death observed in the cultures of transfected COS-7.

### Effect of the N279K Mutation on Tau Phosphorylation in COS-7 Cells

**Figure 3:**
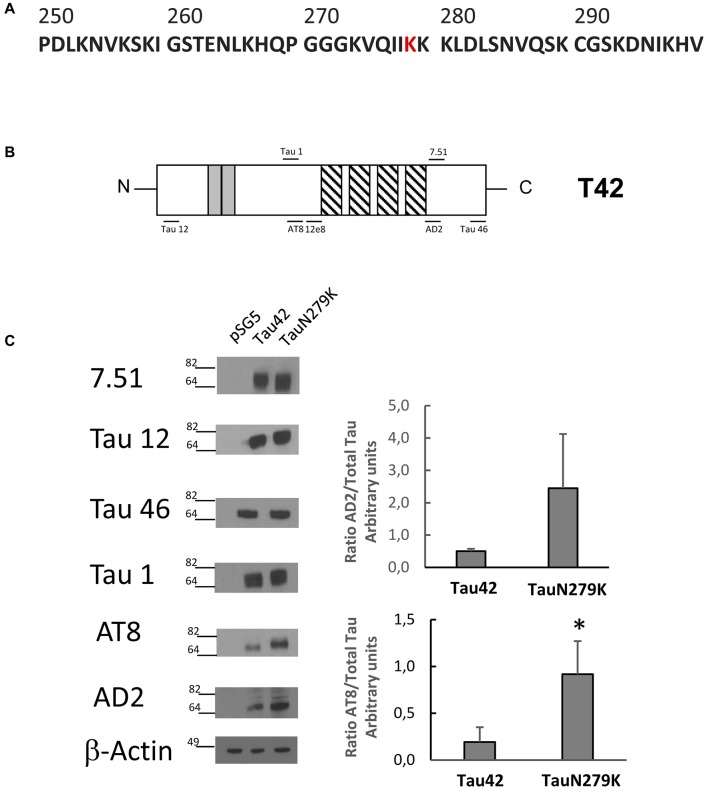
Analysis of human Tau 42 and Tau N279K mutant in COS-7 cells. **(A)** Human Tau 250–300 sequence showing mutated amino acid N279K in red. **(B)** Schematic representation of Tau 42 (T42) correspond to the longest human cDNA isoform of the protein (Goedert et al., 1989). Gray squares indicate the 29-amino acid inserts close to the N-terminal end. Striped square indicates the tubulin binding repeats. Epitopes of antibodies used are indicated. **(C)** Wild-type Tau (Tau 42) and mutant Tau (Tau N279K) were transfected in COS-7 cells. Forty-eight hours after transfection, cell lysates were obtained and analyzed by Western-blot using several anti-Tau antibodies. Actin amount was used in each case as protein loading control. To analyze phosphorylation at AT8 epitope, cells were incubated during 1 h with okadaic acid (0.5 μM). Quantification of AT8 and AD2 antibodies are shown using 7.51 antibody to measure total unphosphorylated tau. AD2 quantification did not show a significant difference, although a tendency toward increase was found. Data are mean ± SD from four separate experiments (*p < 0.05).

The human wild-type Tau 42 and mutant Tau N279K (Figure 3A) plasmids were transfected into COS-7 cells to explore the effect of the N279K mutation on Tau phosphorylation. The level of Tau was determined by measuring the reactivity of the protein with the monoclonal antibodies T12, T46, 7.51 and Tau 1, which recognize Tau independently of its modification state, and AT8 and AD2, which recognizes phosphoserine 202 and phosphoserines 396/404 respectively (Buée-Scherrer et al., 1996; Figure 3B). AT8 antibody did not recognize phosphorylated human wild-type Tau 42 or mutant Tau N279K (data not shown). However, when transfected cells were incubated during 1 h with okadaic acid (0.5 μM) an increase in phosphorylated tau could be observed. Figure 3C shows an increase in the level of phosphorylation at AT-8 epitope of the N279K mutated Tau protein compared with that of wild-type Tau. AD2 signal did not increase at significant levels although an increasing trend was observed. *In vitro* studies have shown that the N279K mutation does not alter the binding of Tau to MTs or decrease the ability of Tau to promote MT assembly (Hong et al., 1998; Barghorn et al., 2000), our results would suggest that phosphorylation of Tau N279K at AT8 epitope could bind to microtubules worse than wild-type Tau.

### Effect of the N279K Mutation on Nuclear Localization of Tau in COS-7 Cells

**Figure 4:**
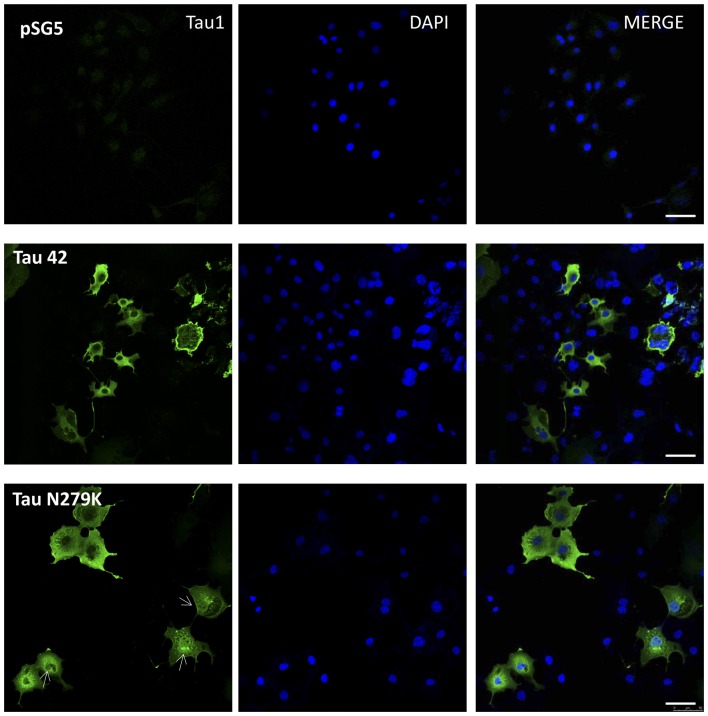
Localization of Tau in COS-7 cells transfected with empty plasmid pSG5, human Tau 42 or Tau N279K. Representative images of localization of Tau 42 and Tau N279K using anti-Tau 1 antibody (green). The localization of nucleus was determined by immunofluorescence with 4′,6-Diamidine-2′-phenylindole dihydrochloride (DAPI). Bar indicates 50 μm. Arrows show nuclear localization of transfected Tau.

**Figure 5:**
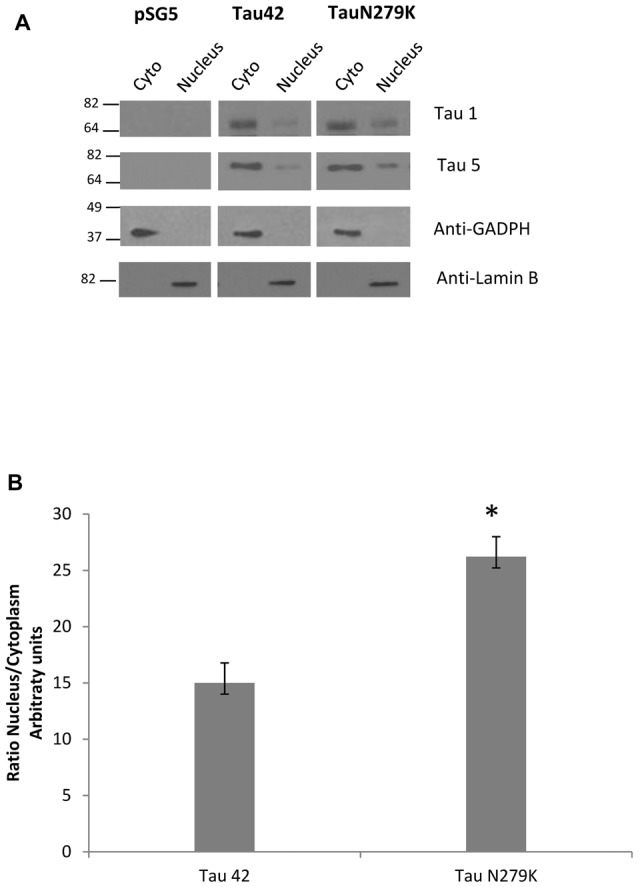
Analysis of Tau localization by Western-blot. **(A)** Representative Western-blot analysis using unphospho-dependent Tau antibody Tau 1 and Tau5. COS-7 cells transfected with both Tau constructs and empty plasmid were subjected to cellular fractionation as described in “Materials and Methods” section. Distribution of Tau was analyzed in cytosolic (Cyto) and nuclear (nucleus) fractions. GADPH and Lamin B were respectively used as specific markers of the cytoplasmic and the nuclear fractions. **(B)** Quantification of ratio Nucleus/Cytoplasm of Tau-1 signal is the mean ± SD (*n* = 4, *p < 0.05).

Many studies have indicated that Tau is present in both the cytoplasmic and nuclear compartments (see review Bukar Maina et al., 2016). To analyze the subcellular distribution of Tau N279K, we first performed analysis by confocal microscopy for cytoplasmic or nuclear localization of Tau constructs. While Tau 42 showed a preferential cytoplasmic localization, Tau N279K was present in both the cytoplasm and the nucleus (Figure 4). To confirm these data obtained by immunofluorescence, we performed subcellular fractionation (see “Materials and Methods” section) on transient transfected COS-7 cells with both plasmids and then analyzed by Western blot analysis with Tau1 antibody (Figure 5A). We used anti-GADPH antibody as a cytoplasm marker antibody and the anti-Lamin B1 antibody as a nuclear marker antibody. Tau 42 can be found in the cytosol compartment as well as in the nuclear compartment. This was in agreement with previous results obtained in human neuroblastoma cells (Loomis et al., 1990). Also, we detected Tau N279K in either cytosol as in nucleus fraction, being the levels of Tau N279K in the nucleus slight greater than Tau 42 (approximately it was increased up to 1.5-fold, Figure 5B). This result would suggest a role of N279K in subcellular localization of Tau in COS-7 cells.

**Figure 6:**
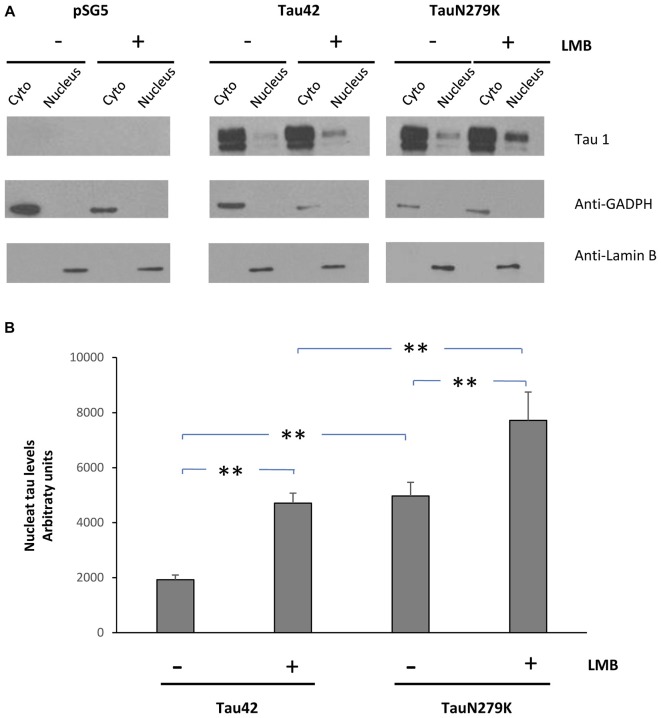
Treatment of cells with an inhibitor of nuclear export, LMB, causes retention of Tau in the nucleus. **(A)** COS-7 cells transfected with empty plasmid, Tau 42 or Tau N279K were incubated with LMB for 1 h. Distribution of Tau was analyzed in cytosolic (Cyto) and nuclear (nucleus) fractions by Western-blot using Tau 1 antibody. GADPH and Lamin B were respectively used as specific markers of the cytoplasmic and the nuclear fractions. **(B)** Quantification of the nucleus intensity of Tau 42 and Tau N279K are shown. Data are mean ± SD (*n* = 3; **p < 0.01).

To confirm that the mutation N279K localizes in the nuclear compartment, we treated COS-7 cells with Leptomycin an inhibitor of nuclear export that blocks the transport of the nuclear export signals (NES)-containing protein from the nucleus to the cytoplasm (Kudo et al., 1998). COS-7 cells were transfected with each of the constructs and cytoplasmic–nuclear Tau localization was analyzed by Western blot (Figure 6A). The accumulation of both Tau 42 and Tau N279K was approximately increased up to 2-fold in the nucleus of COS-7 cells after cell treatment with Leptomycin B (Figure 4B). Furthermore, transfection of mutant Tau into the cells resulted in a greater nuclear localization respect Tau 42 (Figure 6B).

Taken together, data presented in this work suggest that the Tau mutation N279K alters distribution between the cytosol and the nucleus and likely contribute to neurodegeneration.

## Discussion

Tau residue N279 has been extensively studied. Thus, this asparagine can be modified by deamination in Alzheimer disease samples (Dan et al., 2013) and it may favor Tau aggregation (Montejo de Garcini et al., 1986). Asparagine-279 can be mutated to lysine causing one of the most frequent causes of familial frontotemporal dementia. That mutation increases Tau4R/Tau3R ratio by altering alternative splicing of exon 10 (Delisle et al., 1999), but has not effects on Tau binding to microtubules or the ability of Tau to promote MT assembly (Hong et al., 1998). In this work, we have analyzed the consequences of that mutation on the subcellular localization of Tau protein.

Our results demonstrate by biochemical and immunohistochemistry studies that N279K mutation alters distribution between the cytosol and the nucleus. We have found that human Tau bearing the mutation N279K is located, in higher proportion than wild-type Tau, at the cell nucleus. Taking into account this, and that NES are domains rich in leucines, we observed that two leucines are found in position 282 and 284. Thus, we wonder if there exist a nuclear export sequence around that epitope. Bioinformatic analysis of all Tau sequence using the neuronal network/hidden Markow model-based prediction method NetNES^2^ (la Cour et al., 2004) predicts a NES between residues 277 and 284 in the case of mutant protein. The calculated “NES score” exceeds the threshold between residues 277 and 284 (IIKKKLDL) while wild-type sequence (IINKKLDL) the threshold is only exceeded by the L284 suggesting that N279K mutation participates in generate a stronger nuclear export signal. Interestingly all the sequence is present in exon 10 confirming experimental data suggesting that Tau4R isoforms are found in the nucleus (Liu and Götz, 2013). However, while *in silico* data propose that N279K mutation creates a stronger NES in exon 10, our biochemical data suggested that this is not the case. In fact, overexpression of mutant Tau results in the opposite, an increase in the proportion of mutant Tau presents in the nucleus. In any case, further investigations are necessary in order to know if the export/import nuclear machinery and Tau phosphorylation present in COS-7 cells are different compared with neuronal cells or if the system is saturated in overexpressing cells. It should be taken into account that, as has been recently published (Eftekharzadeh et al., 2017), phosphorylated Tau may disrupt nucleocytoplasmic transport through the binding of phosphoTau to nuclear pore machinery.

We here also show that one of the characteristics of N279K Tau is that is highly phosphorylated compared with wild-type Tau as determined by its interaction with antibody AT8 and AD2. The result of that phosphorylation could be a dysfunction at the nuclear pore, this together with the fact that cytoplasmic aggregates can disrupt nucleocytoplasmic transport of proteins and RNAs (Woerner et al., 2016) could explain to some extent the toxicity of N287K Tau. An additional explanation to the results obtained would be that taking into account that phosphorylated Tau binds less to microtubules than the unphosphorylated Tau, it is possible that more Tau would be available for redistributing into the nuclear compartment.

It is also possible that other Tau domains could be involved in nuclear Tau localization. Thus, in the adult murine brain, Tau mainly have four microtubule-binding repeats and the presence of only one amino-terminal inserts in the 4R isoform are localized in the nucleus while those 4R isoforms with 0 or 1 amino-terminal inserts N-terminal are hardly localized in the nucleus (Liu and Götz, 2013).

In summary, we like to support the role of exon 10 (Tau 4R) for the presence of Tau at the cell nucleus, a role that could be facilitate by the presence of N279K Tau mutation.

## Author Contributions

JA, FH and MP conceived and designed the experiments. MR, VG-E and MP performed experiments, analyzed and discussed results. JA, FH and MP wrote the manuscript with feedback from all authors.

## Conflict of Interest Statement

The authors declare that the research was conducted in the absence of any commercial or financial relationships that could be construed as a potential conflict of interest.

## Footnotes

^1^https://www.alzforum.org/mutation/mapt-n279k

^2^http://www.cbs.dtu.dk/services/NetNES/
